# Drug discovery for neutrophil-mediated inflammation

**Published:** 2014-01

Neutrophils are key cells of the innate immune system, the first line of defense against infection. However, neutrophils can drive a chronic inflammatory response that underlies the pathogenesis of many common diseases, including chronic obstructive pulmonary disease and inflammatory bowel disease. Despite the prominence of neutrophil-mediated inflammation in disease, there are no suitable neutrophil-targeted treatments currently available. A new report from the laboratories of Stephen Renshaw and Philip Ingham describes a novel approach for the rapid identification of compounds that can inhibit neutrophil recruitment in response to tissue injury *in vivo*. Using a zebrafish line in which neutrophils are labelled by GFP expression, the team screened a library of fungi-derived compounds and pinpointed a mycotoxin and a natural antibiotic with specific inhibitory effects on neutrophil migration. An *in vivo* imaging assay showed that the antibiotic acts independently of commonly implicated signalling pathways, suggesting an unanticipated mechanism of action. This work demonstrates that zebrafish can be used as a robust *in vivo* platform for high-throughput drug discovery. Page 163

